# Distribution and Functional Analysis of Isocitrate Dehydrogenases across Kinetoplastids

**DOI:** 10.1093/gbe/evae042

**Published:** 2024-03-06

**Authors:** Ľubomíra Chmelová, Kristína Záhonová, Amanda T S Albanaz, Liudmyla Hrebenyk, Anton Horváth, Vyacheslav Yurchenko, Ingrid Škodová-Sveráková

**Affiliations:** Life Science Research Centre, Faculty of Science, University of Ostrava, Ostrava, Czechia; Life Science Research Centre, Faculty of Science, University of Ostrava, Ostrava, Czechia; Institute of Parasitology, Biology Centre, Czech Academy of Sciences, České Budějovice, Czechia; Department of Parasitology, Faculty of Science, Charles University, BIOCEV, Vestec, Czechia; Division of Infectious Diseases, Department of Medicine, Faculty of Medicine and Dentistry, University of Alberta, Edmonton, Canada; Life Science Research Centre, Faculty of Science, University of Ostrava, Ostrava, Czechia; Life Science Research Centre, Faculty of Science, University of Ostrava, Ostrava, Czechia; Department of Biochemistry, Faculty of Natural Sciences, Comenius University, Bratislava, Slovakia; Life Science Research Centre, Faculty of Science, University of Ostrava, Ostrava, Czechia; Life Science Research Centre, Faculty of Science, University of Ostrava, Ostrava, Czechia; Institute of Parasitology, Biology Centre, Czech Academy of Sciences, České Budějovice, Czechia; Department of Biochemistry, Faculty of Natural Sciences, Comenius University, Bratislava, Slovakia

**Keywords:** TCA cycle, Krebs cycle, isocitrate dehydrogenase, NADP^+^, NAD^+^, cofactor preference

## Abstract

Isocitrate dehydrogenase is an enzyme converting isocitrate to α-ketoglutarate in the canonical tricarboxylic acid (TCA) cycle. There are three different types of isocitrate dehydrogenase documented in eukaryotes. Our study points out the complex evolutionary history of isocitrate dehydrogenases across kinetoplastids, where the common ancestor of Trypanosomatidae and Bodonidae was equipped with two isoforms of the isocitrate dehydrogenase enzyme: the NADP^+^-dependent isocitrate dehydrogenase 1 with possibly dual localization in the cytosol and mitochondrion and NADP^+^-dependent mitochondrial isocitrate dehydrogenase 2. In the extant trypanosomatids, isocitrate dehydrogenase 1 is present only in a few species suggesting that it was lost upon separation of *Trypanosoma* spp. and replaced by the mainly NADP^+^-dependent cytosolic isocitrate dehydrogenase 3 of bacterial origin in all the derived lineages. In this study, we experimentally demonstrate that the omnipresent isocitrate dehydrogenase 2 has a dual localization in both mitochondrion and cytosol in at least four species that possess only this isoform. The apparent lack of the NAD^+^-dependent isocitrate dehydrogenase activity in trypanosomatid mitochondrion provides further support to the existence of the noncanonical TCA cycle across trypanosomatids and the bidirectional activity of isocitrate dehydrogenase 3 when operating with NADP^+^ cofactor instead of NAD^+^. This observation can be extended to all 17 species analyzed in this study, except for *Leishmania mexicana*, which showed only low isocitrate dehydrogenase activity in the cytosol. The variability in isocitrate oxidation capacity among species may reflect the distinct metabolic strategies and needs for reduced cofactors in particular environments.

**Significance**

The evolutionary history complex of isocitrate dehydrogenase in Euglenozoa is entangled. Here, we map the distribution of these enzymes in all available kinetoplastid genomes, experimentally test their cofactor preference, and biochemically determine their localization in a set of selected trypanosomatids. We concluded that similarities in mitochondrial and glycosomal metabolism between species of Trypanosomatidae do not always correlate with their phylogenetic relatedness implying that these traits might be shaped by convergent evolution.

## Introduction

Trypanosomatids are a diverse, widespread, and ecologically important group of unicellular eukaryotes, i.e. protists, belonging to the class Kinetoplastea (Kostygov et al. 2021, 2024). Most representatives of this group are monoxenous species (their life cycle is restricted to a single host, usually an insect) (Frolov et al. 2021). Conversely, dixenous species alternate hosts usually shuttling between leeches or insects and vertebrates or plants (Stuart et al. 2008; Lukeš et al. 2014).

The biochemistry of trypanosomatids reflects conditions in the ecological niche they occupy and the specificity of the substrates available for parasites’ growth and differentiation. Moreover, dixenous species must cope with the conditions of two different hosts. As such, they heavily rely on metabolic adaptation to different carbon sources, temperatures, and other factors (Bringaud et al. 2006; Michels et al. 2021). The genome comparison among kinetoplastids revealed that the genetic background for canonical metabolism in this group is similar with just a few exceptions of gene losses and gains that occurred independently several times in the evolution of these parasites (Porcel et al. 2014; Verner et al. 2014; Škodová-Sveráková et al. 2015; Jackson et al. 2016; Opperdoes et al. 2016). However, the identification of a gene in the genome does not automatically imply that its enzymatic product is functionally involved in the metabolism of a given species (Coustou et al. 2003; Saunders et al. 2011).

Isocitrate dehydrogenases (IDHs) are broadly distributed enzymes that, among trypanosomatids, have been mostly studied in dixenous parasites of vertebrates and plants, but not in the group as a whole (Fernández-Ramos et al. 1999; Leroux et al. 2011; Giordana and Nowicki 2020). The IDHs catalyze oxidative decarboxylation of isocitrate into α-ketoglutarate (2-oxoglutarate) with the concomitant release of CO_2_ and the reduction of the cofactor NAD^+^ or NADP^+^. Most prokaryotes possess only NADP^+^-dependent IDHs, while eukaryotes can have both NADP^+^-dependent (IDH1 and IDH2) and NAD^+^-dependent (IDH3) enzymes (Spaans et al. 2015). The cofactor preference usually governs the localization of the enzyme within the cell. Whereas IDH1 and IDH2 operate in both cytosol and mitochondria, IDH3 was considered strictly mitochondrial enzyme involved in the Krebs or tricarboxylic acid (TCA) cycle (Cavalcanti et al. 2014). Moreover, in fungi, plants, and various protists, IDH isoenzymes may localize in other organelles, such as plastids, peroxisomes, or their specialized versions, called glycosomes (Corpas et al. 1999; Vinekar et al. 2012). While in model eukaryotes, IDH3 produces NADH to supply the respiratory chain via complex I and to facilitate ATP synthesis via complex V (Qi et al. 2008), IDH1 and IDH2 generate NADPH needed for the synthesis of nucleotides, fatty acids, or cholesterol and cell protection against the redox stress (Jo et al. 2001; Koh et al. 2004). It is presumed that NAD^+^ dependency is an ancestral trait and the later switch to NADP^+^ is an adaptive evolutionary event enabling bacteria to survive in the energy-poor environment (Hurley et al. 1996; Wang et al. 2015). As such, the cofactor preference of enzymes is crucial for ensuring the proper regulation of metabolism. All IDH3s described so far are regulated allosterically (Lin and McAlister-Henn 2003; Chen et al. 2022). Conversely, in *Escherichia coli*, IDH1 and IDH2 are regulated by the phosphorylation of a single Ser in the active site preventing the isocitrate binding (Dean et al. 1989), whereas in mammals, the same amino acid interacts with a conserved Asp providing a feedback loop for self-regulation (Xu et al. 2004).

The genomes of *Trypanosoma cruzi* and *Trypanosoma brucei*, for which employment of a noncanonical TCA cycle has been reported (van Weelden et al. 2003; Villafraz et al. 2021), encode two putative IDHs (IDH1 and IDH2). In *T. cruzi*, both enzymes were shown to depend on NADP^+^ (Leroux et al. 2011), while IDH1 of *T. brucei* showed activity with both NAD^+^ and NADP^+^ cofactors (Wang et al. 2017). IDH1 of *Trypanosoma* spp. possesses a possible peroxisomal targeting signal 1 (van Weelden et al. 2005; Colasante et al. 2006) and was recently experimentally shown to localize to both mitochondrion and cytosol in *T. brucei* (Pyrih et al. 2023). Complicating the situation even more, both the NADP^+^-dependent (related to IDH2) and NAD^+^-dependent (related to IDH3) isoforms with dual mitochondrial and cytosolic localization were described in *Leishmania mexicana* (Giordana and Nowicki 2020).

In this study, we map the distribution of IDH enzymes in all available kinetoplastid genomes, experimentally test their cofactor preference, and biochemically determine their localization in a set of selected trypanosomatids.

## Results

### Distribution and Origin of IDHs

**Fig. 1.**
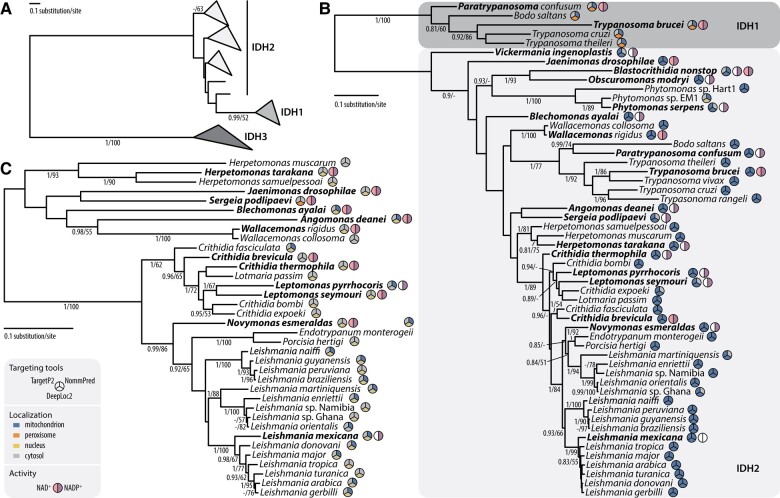
Phylogenetic analysis of A) all identified IDH proteins, B) IDH1 + 2, and C) IDH3 of kinetoplastids. MrBayes topology of phylogenetic trees is shown, onto which posterior probabilities (PP) and ultrafast bootstrap support (UFB) values from IQ-TREE are overlayed. Only support values PP ≥ 0.8 and UFB ≥ 50% are shown. Protein localization prediction and measured IDH activities are shown in circles as explained in the graphical legend. Protein localization predictions were performed for all sequences (supplementary table S1, Supplementary Material online), whereas IDH activities were measured for species in bold (supplementary table S2, Supplementary Material online). In case of *Blastocrithidia nonstop*, only one sequence was identified but different activities were measured in mitochondrial and cytosolic fractions; therefore, two colored circles are shown. Note the data from *Paratrypanosoma* sp. EC233 and *Wallacemonas raviniae* are unavailable or very fragmented; therefore, for phylogenetic analyses, *P. confusum* and *W. rigidus*/*W. collosoma* were used instead (only species names are in bold in these cases). For full trees in Newick format and alignments, see supplementary Data S1 to S3, Supplementary Material online.

We identified 90 IDH sequences in 49 kinetoplastid data sets (supplementary table S1, Supplementary Material online). To determine their affiliation to a specific class (IDH1, 2, or 3), we conducted a kinetoplastid-specific phylogenetic analysis (Fig. 1A). IDH1 was identified only in *Bodo saltans*, *Paratrypanosoma confusum*, *T. brucei*, *T. cruzi*, and *Trypanosoma theileri*. Conversely, IDH2 was omnipresent and IDH3 was found in a majority of species with the exception of representatives of genera *Trypanosoma*, *Phytomonas*, and *Paratrypanosoma* (Fig. 1B and C). Coexistence of all three IDHs was not documented in any of the studied kinetoplastids. Our targeting predictions revealed that IDH2s and IDH3s are likely mitochondrial and cytosolic, respectively (Fig. 1; supplementary table S1, Supplementary Material online). However, localization predictions for IDH1s differed depending on the employed tool suggesting a dual localization of this isoenzyme.

**Fig. 2.**
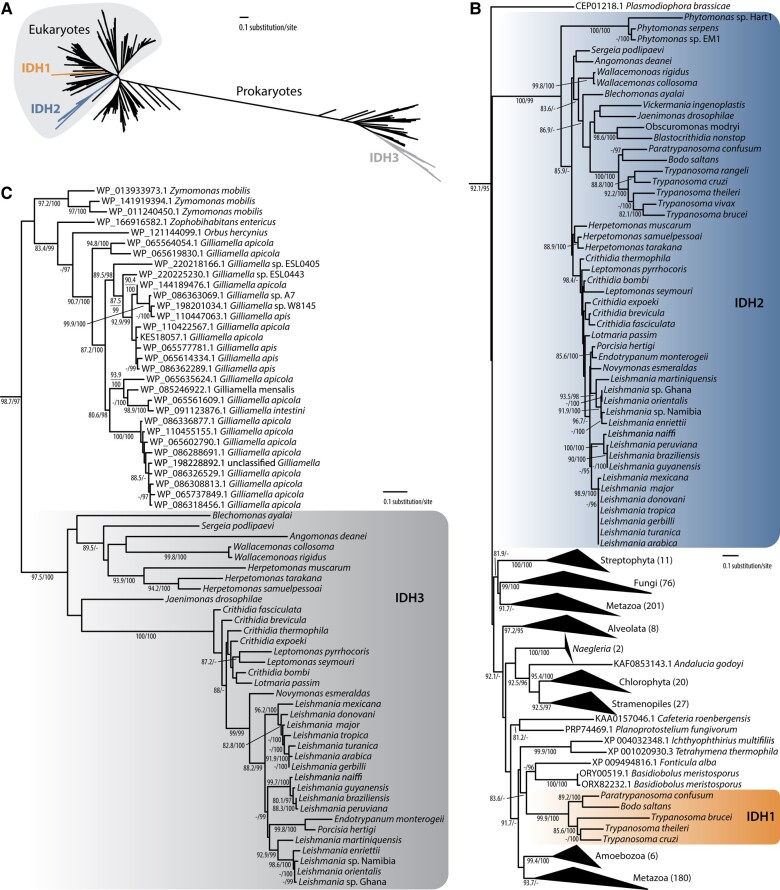
Phylogenetic analysis of IDH proteins. A) Unrooted full tree (see supplementary Data S4, Supplementary Material online for the tree in Newick format and alignment). B) Part of the tree encompassing kinetoplastids' IDH1 and IDH2. C) Part of the tree encompassing kinetoplastids' IDH3. Support values are shown as SH-aLRT and ultrafast bootstrap supports if ≥80% and ≥95%, respectively.

To investigate origin and evolutionary history of IDH enzymes in kinetoplastids, we performed more comprehensive phylogenetic analysis including other eukaryotes and prokaryotes (Fig. 2). Whereas IDH2 is presumably an ancestral eukaryotic ortholog of IDH, IDH1 and IDH3 appear to be acquired by lateral gene transfer from eukaryotes and bacteria by the common ancestor of kinetoplastids and after the split of *Trypanosoma* spp., respectively. IDH1 was nested in a large clade of obazoans and ciliates, and IDH3 was gained from an unclear source of the Proteobacteria.

### NADP^+^ Preference for IDH Activity

**Fig. 3.**
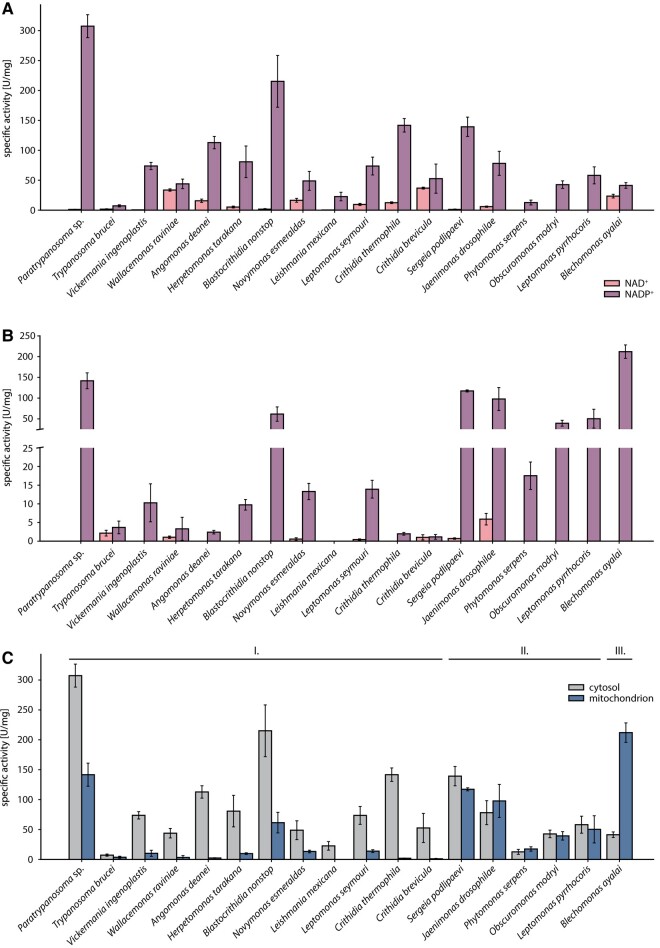
IDH cofactor specificity and activity distribution. Specific IDH activity in A) cytosolic and B) mitochondrial fraction with NAD^+^ and NADP^+^ cofactors. C) Comparison of NADP^+^-dependent IDH activity between cytosol and mitochondrion. Groups I, II, and III refer to species with elevated NADPH generation in cytosol (labeled I.), similar production of NADPH in both compartments (labeled II.), and elevated NADPH generation in mitochondrion (labeled III.). 1 U = nmol of NADH/NADPH per min per milligram of total protein.

Firstly, we measured the IDH activity in cytosolic fractions of selected trypanosomatids with both cofactors (NADP^+^ and NAD^+^) (Fig. 3A; supplementary table S2, Supplementary Material online). Only in the case of *Wallacemonas raviniae*, the choice of cofactor did not affect activity (33.6 ± 1.9 U/mg for NAD^+^ and 43.9 ± 7.9 U/mg for NADP^+^). In 12 other species (*Angomonas deanei*, *Blechomonas ayalai*, *Blastocrithidia nonstop*, *Crithidia brevicula*, *Crithidia thermophila*, *Herpetomonas tarakana*, *Jaenimonas drosophilae*, *Leptomonas seymouri*, *Novymonas esmeraldas*, *Paratrypanosoma* sp., *Sergeia podlipaevi*, and *T. brucei*), we detected activities with both cofactors, but NADP^+^ was strongly preferred. In the rest of our data set (five strains), the enzymatic activity was not detected with NAD^+^. Activities of NAD^+^-dependent IDH in mitochondrial fraction were either not detectable or low (under 1 U/mg) for all tested trypanosomatids except for *C. brevicula* (1.0 ± 0.7 U/mg), *J. drosophilae* (5.9 ± 1.6 U/mg), *T. brucei* (2.1 ± 0.8 U/mg), and *W. raviniae* (1.0 ± 0.3 U/mg) (Fig. 3B; supplementary table S2, Supplementary Material online).

### Distribution of IDH Activity in the Cell

Since NAD^+^ does not appear to be a main cofactor for IDH activities neither in cytosol nor in mitochondrion of trypanosomatids, the comparison between cellular compartments was made only with NADP^+^ (Fig. 3C; supplementary table S2, Supplementary Material online). We recorded lower NADP^+^-dependent IDH activity in the mitochondrion when compared to the cytosol for *A. deanei*, *B. nonstop*, *C. brevicula*, *C. thermophila*, *H. tarakana*, *L. mexicana, L. seymouri*, *N. esmeraldas*, *Paratrypanosoma* sp., *Vickermania ingenoplastis*, and *W. raviniae*. Some species (*J. drosophilae*, *Leptomonas pyrrhocoris*, *Obscuromonas modryi*, *Phytomonas serpens*, *S. podlipaevi*, and *T. brucei*) showed comparable NADP^+^-dependent IDH activities in both fractions. The only species showing higher NADP^+^-dependent IDH activity in mitochondrion than in cytosol was *B. ayalai*.

### Dual Localization of IDH2

Possible dual localization of IDH2 was indicated by two observations. Firstly, *B. nonstop*, *O. modryi*, *P. serpens*, and *V. ingenoplastis* bear only one copy of IDH enzyme (specifically, IDH2) unambiguously predicted to mitochondrion in *O. modryi*, but to mitochondrion and cytosol in the remaining species (Fig. 1; supplementary table S1, Supplementary Material online). In the latter organisms, one enzyme appears to operate in two compartments (Fig. 3C). The purity of fractions was also checked to ensure no cross-contamination (supplementary fig. S1, Supplementary Material online). We cannot rule out the dual localization of IDH2 in other trypanosomatids (Fig. 1), but the presence of either IDH1 or IDH3 makes it impossible to unambiguously assign the cytosolic activity in these species. Secondly, although NAD^+^-dependent mitochondrial IDH3 is absent in *T. brucei*, we found low activities with both cofactors in the mitochondrial fraction (Fig. 3B). This could be explained by the activity of IDH2 with dual specificity for cofactors. In the whole data set (except for *L. mexicana*), we determined mitochondrial NADP^+^-dependent IDH activity that can be attributed to IDH2 (Fig. 3B; supplementary table S2, Supplementary Material online).

## Discussion

In this work, we present a comprehensive analysis of IDH enzymes in kinetoplastids and demonstrate their rather complex evolutionary history. The most parsimonious explanation of the existing data is that the last common ancestor of Trypanosomatidae and Bodonidae was equipped with two isoforms of this enzyme (NADP^+^-dependent IDH1 with possibly dual localization in the cytosol and mitochondrion and NADP^+^-dependent mitochondrial IDH2) following the loss of mitochondrial NAD^+^-dependent IDH3, which is present in other related taxa. While IDH2 was retained in the evolution of trypanosomatids, IDH1 was lost upon separation of *Trypanosoma* spp. (Skalický et al. 2017) and replaced by the mainly NADP^+^-dependent cytosolic IDH3 of proteobacterial origin in all the derived lineages. The bacterial origin of IDH3 has been already suggested before (Andrade-Alviárez et al. 2022). In the same paper, a different evolutionary pattern of IDH enzymes was described for the sister group of diplonemids, in which the loss of IDH2 was compensated by the duplication of IDH1.

We have observed different distributions of NADP^+^-dependent IDH activities in the studied species (Fig. 3C): (I) elevated conversion of NADP^+^ into NADPH in the cytosol, (II) similar levels of NADP^+^ conversion in both compartments, and (III) elevated conversion of NADP^+^ into NADPH in the mitochondrion. This could reflect different metabolic needs and compartmentalization of the enzyme (Lewis et al. 2014; Kovářová and Barrett 2016).

The data on generally low or absent NAD^+^-dependent IDH activities in kinetoplastids from this study agree with previous results (Meade et al. 1984; Durieux et al. 1991) and further confirm the existence of a noncanonical TCA cycle in Trypanosomatidae (van Weelden et al. 2003; Besteiro et al. 2005). Notably, *Leishmania* promastigotes preferentially catabolize glucose via glycolysis, succinate fermentation, and a full TCA cycle (Louassini et al. 1999; Saunders et al. 2014) implying that the TCA cycle is not a fixed pathway, as was thought at the times of its discovery, but it can be rerouted in response to the changing environmental cues (Lane 2022). The TCA cycle in the studied species is actually noncyclic because of the low activities of aconitase and IDH in comparison with other TCA enzymes leading to diversion of the metabolites by more active enzymes (van Hellemond et al. 2005). In line with this, the accumulation of reactive oxygen species in mitochondria was recently associated with an increased NADPH/NADH ratio and the buildup of 2-hydroxyglutarate, a metabolite of α-ketoglutarate (Xiao et al. 2018). Under changing environmental conditions that many trypanosomatids experience during their life cycle, the reduction of NAD^+^-dependent IDH activity appears metabolically justified. In the presented work, the only deviation from the strict NADP^+^ dependence of mitochondrial IDH activity was documented in *J. drosophilae* making this underinvestigated species a good candidate for further metabolomic studies. Conspicuously, overexpressed and biochemically purified *T. brucei* and *L. mexicana* IDHs demonstrated dual cofactor specificity (Wang et al. 2017; Giordana and Nowicki 2020), but the data presented here suggest that the NAD^+^ dependence of these enzymes is likely to be an in vitro artifact.

Taking together, our results on metabolic variability across Trypanosomatidae warrant future follow-up studies in this important group of eukaryotic parasites. Indeed, a previous report published by our group posited that similarities in mitochondrial metabolism between species of Trypanosomatidae do not always correlate with their phylogenetic relatedness implying that these traits might be shaped by convergent evolution (Škodová-Sveráková et al. 2015; Opperdoes et al. 2021). This observation can now be extended onto cytosolic metabolism and exemplified by IDHs. Yet, it remains to be investigated how universal this rule is.

## Materials and Methods

### Sequence Searches and Targeting Predictions

The isocitrate/isopropylmalate dehydrogenase hidden Markov model profile PF00180 from the Pfam v. 36.0 database (Mistry et al. 2021) was used for HMMER v. 3.3.2 (Eddy 2011) search in the genome-derived protein data sets of kinetoplastids (Albanaz et al. 2023; Kostygov et al. 2024). The search retrieved 62 proteins, which were then used as queries in tBLASTn v. 2.13.0 (Camacho et al. 2009) searches in genomes for which no proteomes were available or in the cases when no protein hit was retrieved. The gene boundaries were determined and the protein sequences were retrieved using Artemis v. 18.1 (Carver et al. 2012). A total of 90 protein sequences from 49 kinetoplastid species were collected (supplementary table S1, Supplementary Material online). The subcellular localization predictions of IDH proteins were performed by TargetP v. 2.0 (Armenteros et al. 2019), NommPred v. 0.3 (Kume et al. 2018), and DeepLoc v. 2.0 (Thumuluri et al. 2022).

### Phylogenetic Analyses

The data set of 90 kinetoplastid IDH sequences (supplementary table S1, Supplementary Material online) was aligned by MAFFT v. 7.508 (Katoh and Standley 2013) using the L-INS-i method. The resulting alignment was trimmed by TrimAl v. 1.4.rev15 (Capella-Gutiérrez et al. 2009) using the -automated1 option optimized for phylogenetic inferences. The Bayesian analysis was performed using MrBayes v. 3.2.7a (Ronquist et al. 2012) under a mixed amino acid model, with 20 million Markov chain Monte Carlo generations and four gamma rate categories. The sampling frequency was set to every 1,000 generations. The first 25% of the runs were discarded as burn-in. The maximum-likelihood phylogenetic analysis was performed in IQ-TREE v. 1.6.12 (Nguyen et al. 2015) applying the posterior mean site frequency method (Wang et al. 2018) and the LG + C20 + F + G model, with the guide tree inferred in default settings except for adding protein mixture models (-madd C10, C20, C30, C40, C50, C60, LG4M, LG4X, LG + C20 + F + G, LG + C40 + F + G, LG + C60 + F + G) to the model selection process using ModelFinder (Kalyaanamoorthy et al. 2017). The LG + C20 + F + G model was selected for the guide tree as the best fitting according to the Bayesian information criterion (Evans and Sullivan 2011). The branch supports were estimated with 1,000 standard bootstrap replicates (Hoang et al. 2018). Bootstrap support values were overlaid onto the MrBayes tree topology with posterior probabilities. To increase the support values at the backbone of the tree, we performed separate phylogenetic analyses for the two main clades (IDH1 + 2 and IDH3) using the same methods and parameters as above, except for inferring the guide tree using the LG + F + G model.

To investigate the origin of kinetoplastid IDH proteins, their sequences were used in BLASTp searches (*e*-value cutoff of 10^−5^) against the NCBI nonredundant database excluding Kinetoplastea taxids. A maximum of 100 hits per sequence was collected. The retrieved 1,413 sequences were deduplicated using MMseqs2 v. 14 (Steinegger and Söding 2017) with a minimum sequence identity of 99% and a minimum bidirectional coverage of 50%. The resulting data set of 958 sequences was added to the kinetoplastid one, aligned, and trimmed as described above. The maximum-likelihood phylogenetic tree was inferred in IQ-TREE using the LG + C20 + F + G model, with 1,000 replicates for ultrafast bootstraps (Hoang et al. 2018) and Shimodaira–Hasegawa approximate likelihood ratio test (SH-aLRT) (Guindon et al. 2010) and a maximum of 5,000 iterations.

### Strains and Cultivation Conditions

*Trypanosoma brucei* 29-13 was grown at 27 °C in SDM-79 (Thermo Fisher Scientific, Waltham, USA) supplemented with 10% (v/v) fetal bovine serum (Biosera, Cholet, France), 2-μg/mL hemin (Merck, Darmstadt, Germany), 50 U/mL penicillin, and 50-μg/mL streptomycin (both from Biowest, Nuaillé, France). *Sergeia podlipaevi* CER4 was grown at 23 °C in BHI medium (VWR/Avantor, Radnor, USA) supplemented as above. *Paratrypanosoma* sp. EC233, *B. ayalai* B08-376, *V. ingenoplastis* CP21, *W. raviniae* ECU-09, *A. deanei* CT-IOC-044, *J. drosophilae* Finn02, *P. serpens* 9T, *H. tarakana* OSR18, *O. modryi* Fi15, *B. nonstop* P57, *N. esmeraldas* E262, *L. mexicana* MNYC/BZ/62/M379, *L. seymouri* ATCC 30220, *L. pyrrhocoris* H10, *C. brevicula* S14, and *C. thermophila* CT-IOC-054 were grown at 23 °C in Schneider's *Drosophila* medium (Biowest) supplemented as above. Cells were collected in the exponential phase of growth (1 to 2 × 10^7^ cells/mL) for all experiments. Species identity was confirmed as in Yurchenko et al. (2016).

### Isolation of Mitochondria-Enriched Fraction

Cells were pelleted (1,000 × *g*, 10 min, 4 °C), washed in ice-cold PBS, and aliquoted. Approximately 5 × 10^8^ cells (1 aliquot) were resuspended in 1.5-mL NET buffer (150 mM NaCl, 100 mM EDTA, and 10 mM Tris-HCl pH 8) and subsequently pelleted at 16,000 × *g* for 10 min at 4 °C. The pellet was resuspended in 1.5-mL DTE buffer (1 mM Tris-HCl pH 7.9 and 1 mM EDTA) and passed through a 25G needle twice, and in the second step 180 μL of 60% (*w*/*v*), sucrose was added. The sample was centrifuged at 16,000 × *g* for 10 min at 4 °C. The pellet was resuspended in 500-μL of STM buffer (250 mM sucrose, 20 mM Tris-HCl pH 7.9, and 2 mM MgCl_2_), and 10 U of DNAse I (Merck) was added. The reaction was incubated for 30 min on ice and stopped with the addition of 500 μL of STE buffer (250 mM sucrose, 20 mM Tris-HCl pH 7.9, and 2 mM EDTA). The sample was pelleted (16,000 × *g*, 10 min, 4 °C), and the pellet was washed twice in the ice-cold STE buffer and stored at −80 °C. The preparation of mitochondria-enriched fraction was performed for all the analyzed strains in three biological replicates.

### Preparation of Mitochondrial Lysate and Western Blotting

Isolated mitochondria-enriched fraction was resuspended in 0.5 M aminocaproic acid (ACA) (AppliChem/ITW, Darmstadt, Germany) and 2% (w/v) dodecylmaltoside (AppliChem/ITW) and kept on ice for 30 min. The lysate was then centrifuged at 20,000 × *g* for 10 min at 4 °C, and the protein concentration was determined by Pierce BCA Protein Assay Kit (Thermo Fisher Scientific).

The purity of mitochondrial fraction was confirmed by immunoblotting of *T. brucei* fractions probed against the glycosomal and mitochondrial proteins triosephosphate isomerase (TIM, glycosomal marker) and heat shock protein 70 (Hsp70, mitochondrial marker) with rabbit polyclonal anti-TIM (Galland et al. 2007) (1:5,000, provided by P. Michels) and mouse monoclonal anti-Hsp70 (Panigrahi et al. 2008) (1:5,000, provided by J. Lukeš) antibodies. Mouse monoclonal anti-α-tubulin antibody (Sigma-Aldrich/Merck, Darmstadt, Germany) diluted 1:10,000 was used for loading control as in Kraeva et al. (2014).

### Preparation of Cytosolic Lysate

Cells were pelleted at 1,000 × *g* for 10 min at 4 °C and washed in ice-cold PBS. The resulting pellet was resuspended in ice-cold 0.5 M ACA. The protein concentration was determined by Pierce BCA Protein Assay Kit, and the cells in ACA were incubated with 0.1 mg of digitonin per 1 mg of total proteins for 4 min at room temperature as in the previously published papers (Castro et al. 2010; Schenk et al. 2021). Lysates were centrifuged at 16,000 × *g* for 5 min at 4 °C, and the resulting supernatants were quantified by Pierce BCA Protein Assay Kit. The cytosolic lysate was prepared for all the analyzed strains in three biological replicates. Please note that under the conditions used, the cytosolic fraction contained small organelles, such as glycosomes. Nevertheless, they are not likely to affect the downstream analyses because, based on the studies in other trypanosomatids (Villafraz et al. 2021; Wargnies et al. 2021), permeabilization of glycosomal membranes requires higher concentrations of digitonin.

### IDH Activity Assay

The IDH activity was measured as in Giordana and Nowicki (2020) as a production of NAD(P)H at 340 nm for 2 min in a reaction buffer containing 75 mM Tris-HCl pH 7.5, 0.5 mM MnCl_2_, and 5 mM isocitrate. The reaction (final volume 200 μL) contained ∼80 μg of total proteins and was initiated by adding of NAD(P)^+^ to the final concentration of 0.5 mM. One unit is equal to 1 nmol of produced NAD(P)H per min per milligram of total protein. All measurements were performed in three biological replicates with at least three technical replicates each.

## Supplementary Material

evae042_Supplementary_Data

## Data Availability

The data underlying this article are available in the article and in its online Supplementary material.
