# Supplementary material for: Distribution and Functional Analysis of Isocitrate Dehydrogenases across Kinetoplastids
Source: Genome Biol Evol. 2024 Mar 6;16(3):evae042. doi: 10.1093/gbe/evae042 (PMC10946238; doi:10.1093/gbe/evae042)
Supplement: evae042_Supplementary_Data [file evae042_supplementary_data.zip › Supplementary information legends.docx]

# Supplementary information

**Fig. S1. Immunoblot analysis of whole-cell lysates (W) and cytosolic fraction (C) of *T. brucei* after digitonin permeabilization.** The blot was probed with anti-HSP70 and anti-TIM antibodies; α-tubulin served as a loading control.

**Table S1. IDH dataset of kinetoplastid enzymes.**

**Table S2. Specific IDH activity in mitochondrial and cytosolic fractions.**

**Data S1. The full IDH tree in Newick format (A) shown in Fig. 1A and corresponding alignment (B).**

**Data S2. The full IDH tree in Newick format (A) shown in Fig. 1B and corresponding alignment (B).**

**Data S3. The full IDH tree in Newick format (A) shown in Fig. 1C and corresponding alignment (B).**

**Data S4. The full IDH tree in Newick format (A) shown in Fig. 2A and corresponding alignment (B).**
