# Supplementary figures and images for: Distribution and Functional Analysis of Isocitrate Dehydrogenases across Kinetoplastids
Source: Genome Biol Evol. 2024 Mar 6;16(3):evae042. doi: 10.1093/gbe/evae042 (PMC10946238; doi:10.1093/gbe/evae042)

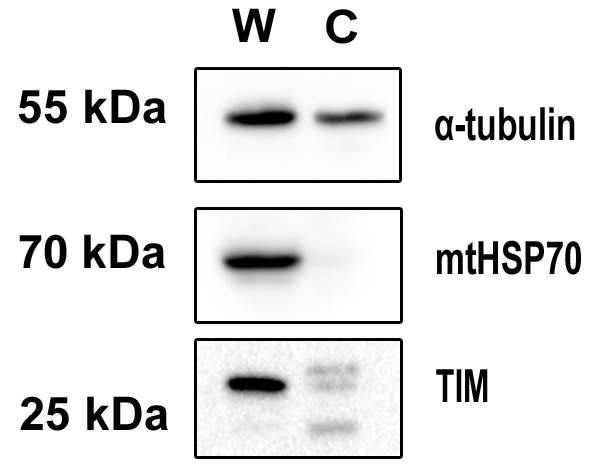

Supplement: evae042_Supplementary_Data [file evae042_supplementary_data.zip › Fig S1 R1.tif]
